# Production and Antimicrobial Activity of Nisin Under Enological Conditions

**DOI:** 10.3389/fmicb.2018.01918

**Published:** 2018-09-05

**Authors:** Rocío Fernández-Pérez, Yolanda Sáenz, Beatriz Rojo-Bezares, Myriam Zarazaga, Juan M. Rodríguez, Carmen Torres, Carmen Tenorio, Fernanda Ruiz-Larrea

**Affiliations:** ^1^Instituto de Ciencias de la Vid y del Vino (Universidad de La Rioja, CSIC, Gobierno de La Rioja), Logroño, Spain; ^2^Area de Microbiología Molecular, Centro de Investigación Biomédica de La Rioja, Logroño, Spain; ^3^Área de Bioquímica y Biología Molecular, Departamento de Agricultura y Alimentación, Universidad de La Rioja, Logroño, Spain; ^4^Department of Nutrition and Food Science, Complutense University of Madrid, Madrid, Spain

**Keywords:** bacteriocin, nisin, wine, sulfur dioxide, winemaking, *Lactococcus lactis*, lactic acid bacteria

## Abstract

Lactic acid bacteria (LAB) are responsible for the malolactic fermentation of wines, and, therefore, controlling the growth of these bacteria is a key factor for elaborating premium wines. Sulfur dioxide has been traditionally used as an efficient antimicrobial and antioxidant agent, however, nowadays consumers’ demand tends toward a reduction of sulfur dioxide levels in wine and other fermented foods. A previous study of our research group had demonstrated the effectiveness of the bacteriocin nisin to inhibit the growth of enological LAB, and its activity had been tested in culture broths. The aim of this study was to investigate the possibility of controlling the growth of bacteria in wine by the use of nisin in combination with sulfur dioxide, and to study nisin production by the natural producer *Lactococcus lactis* LM29 under enological conditions. Our results showed that *L. lactis* LM29 produced nisin in the presence of 2 and 4% ethanol (v/v), while higher concentrations of ethanol fully inhibited the production of nisin. We obtained a nisin enriched active extract (NAE) from the cell-free supernatant of a culture of *L. lactis* LM29 in MRS broth containing 60% (v/v) sterile grape juice, and the extract was fully active in inhibiting the growth of the enological LAB tested by the microtiter method. Moreover, the nisin concentration of the obtained NAE could actually prevent the formation of an undesirable biofilm of LAB strains. Finally, our results of wine ageing under winery conditions showed that the use of 50 mg/L nisin decreased fourfold the concentration of sulfur dioxide required to prevent LAB growth in the wines.

## Introduction

Lactic acid bacteria (LAB) are responsible for malolactic fermentation (MLF) in wine and, therefore, controlling the growth of these bacteria is a key factor for elaborating quality wines (Petruzzi et al., 2017). Currently, MLF can be carried out either spontaneously by indigenous LAB, or by inoculated commercial starters. Sulfur dioxide has been traditionally used as the chemical preservative to control MLF and to prevent the growth of undesirable bacteria in wine and as an efficient antimicrobial and antioxidant agent to prevent wine browning and loss of quality (García-Guzmán et al., 2015). However, nowadays consumers’ demand tends toward a reduction of sulfur dioxide levels in wine and other fermented foods and total sulfur levels are limited (EU regulations 479/2008 and 1129/2011). Expert wine-makers require powerful tools that allow controlling microbial growth at critical stages along the wine-making process, from grape harvesting to wine storage and aging in bottles, to produce flavorsome and premium wines.

Bacteriocins are antimicrobial peptides secreted by certain bacterial strains, which are immune to them, to compete by inhibiting the growth of other bacteria present in their environment. Nisin is a 35-mer bacteriocin of the lantibiotics group, whose use as a food preservative was approved by the European Union (food additive number E234, EEC, 1983) and the Joint Food and Agriculture Organization/World Health Organization (FAO/WHO), and it is currently the most studied and characterized of all the bacteriocins produced by LAB (Smith et al., 2016; EFSA, 2017).

A previous study of our research group demonstrated the effectiveness of nisin to inhibit the growth of LAB of enological origin and its activity was tested by the microtiter method in culture broths (Rojo-Bezares et al., 2007b). The aim of this study was to investigate the possibility of controlling the growth of bacteria in wine by the use of nisin in combination with sulfur dioxide, and to study nisin production by the natural producer *Lactococcus lactis* LM29 under enological conditions.

## Materials and Methods

### Strains and Culture Conditions

The strains used in this study are shown in **Table 1**. Three of them were obtained from the Spanish Type Culture Collection (CECT). The remaining acetic acid bacteria (AAB) of this study were isolated from either wine or vinegar, and the remaining LAB strains were isolated from grape wines and musts, with the exception of the nisin-producing strain *L. lactis* LM29, which was originally isolated from human milk during a previous study (Jiménez et al., 2008). The production of nisin by *L. lactis* LM29 reaches values of, approximately, 2,030 IU/mL (∼41 μg/mL) when grown in MRS broth at 32°C overnight (unpublished data).

**Table 1:** Bacterial strains included in this study.

	Species	Number of strains	Strain name	Origin
LAB^∗^ strains (*n* = 43)	*Lactococcus lactis*	1	LM29	Human milk
	*Pediococcus pentosaceus*	4	FBB63, J27, J29, J40	Enological (UR)
	*Lactobacillus fermentum*	1	C533	Enological (UR)
	*Lactobacillus plantarum*	27	E14, E3, J23, J31, J36, J39, J51, J52, J53, J55, J56, J58, J59, J61, J62, J63, J65, J67, J69, J71, J73, J75, J77, J78, T20, T53, Y17	Enological (UR)
	*Pediococcus acidilactici*	2	IS111, J83	Enological (UR)
	*Leuconostoc mesenteroides*	4	J47, J48, J57, J32	Enological (UR)
	*Lactobacillus brevis*	1	J54	Enological (UR)
	*Lactobacillus hilgardii*	2	J80, J81	Enological (UR)
	*Pediococcus parvulus*	1	J82	Enological (UR)
*O. oeni* (*n* = 38)	*Oenococcus oeni*	38	IS1, IS109, IS11, IS127, IS13, IS135, IS14, IS143, IS151, IS154, IS158, IS159, IS167, IS177, IS18, IS186, IS189, IS196, IS209, IS21, IS24, IS27, IS40, IS41, IS43, IS44, IS45, IS46, IS48, IS51, IS53, IS59, IS73, IS75, IS83, IS92, IS95, IS97	Enological (UR)
AAB strains (*n* = 29)	*Acetobacter aceti*	1	C656	CECT298
	*Gluconobacter oxydans*	9	C657 I34, I38, I40, I7, IS219, IS262, IS283, V3	CECT360 Enological (UR)
	*Acetobacter pasteurianus*	7	C658 IS227, IS245, IS286 R28, R30, R90	CECT474 Enological (UR) Vinegar (UR)
	*Acetobacter orleanensis*	6	IS242, IS259, IS282, IS291, IS293, IS294	Enological (UR)
	*Gluconacetobacter europaeus*	2	R1, R48	Vinegar (UR)
	*Gluconacetobacter xylinus*	4	R35, R36, R41, R46	Vinegar (UR)

*CECT, Spanish Type Culture Collection; UR, strain collection of the University of La Rioja; LAB^∗^, lactic acid bacteria except O. oeni strains; AAB, acetic acid bacteria. Enological, isolated form wines or grape juices.*

Lactic acid bacteria strains were grown on MRS-agar (Scharlau Chemie S.A. Barcelona, Spain) at 30°C under an atmosphere with 5% CO_2_. *Oenococcus oeni* strains were incubated for 72 h and the rest of LAB for 48 h. AAB were grown under aerobic conditions for 48 h at 30°C onto GY agar plates [5% glucose (Panreac Química S.A., Barcelona, Spain), 1% yeast extract (Scharlau Chemie S.A.), and 1.5% agar (Becton Dickinson, Madrid, Spain)].

### Wine Samples and Nisin Treatment

Red wine was elaborated from c.v. *Tempranillo* red grapes from local vineyards of the northern Spanish region of La Rioja, using traditional vinification techniques; i.e., wines were elaborated in the presence of grape skins, seeds, and stalks, with the indigenous yeast strains and after addition of sulfur dioxide. At the end point of alcoholic fermentation, wine was drawn off from the lees and placed homogeneously into fermentation vats (racking) for MLF and the temperature was maintained around 22°C. The chemical composition of the wine was determined according to OIV (2014) official methods, and it was as follows: alcohol 13% v/v; pH 3.8; volatile acidity 0.7 g/L; total acidity 3.0 g/L (as tartaric acid); concentration of total sulfur dioxide 9 mg/L; and turbidity 133 NTUs. Wine was bottled in 750 mL bottles and experiments were carried out in triplicate. Binary combinations of nisin and potassium metabisulphite were tested and 12 different combinations. Commercial nisin (Sigma-Aldrich, Madrid, Spain) was added in the following concentrations, below and above the reported nisin concentration of 12.5 mg/L (Rojo-Bezares et al., 2007b) against wine LAB: 0, 0.78, 6.25, and 50 mg/L (activity ≥10^3^ IU/mg). Potassium metabisulphite (Dolmar, S.L., Spain) was added where necessary to reach total potassium metabisulphite concentrations of 18, 25, and 100 mg/L. One mole of potassium metabisulphite dissolved in wine yields one mole of potassium sulphite and one mole of sulfur dioxide. Wines were allowed to rest under winery conditions (15°C, constant humidity and darkness) for 3 months of aging in glass bottles. After this period, wine samples (10 mL) were taken from each wine and processed as previously described (López et al., 2007). Samples were spun at 3,000 × *g* for 10 min at 4°C (Sorwall RC-5 B Refrigerated Superspeed Centrifuge). Pellets were collected and after appropriate dilutions in sterile saline solution (0.9% NaCl), they were seeded in duplicate onto MRS agar plates with 200 μg of nystatin per mL (Acofarma, S. Coop., Terrassa, Barcelona, Spain). Samples were incubated at 30°C under anaerobic conditions (GasPak, Oxoid Ltd., Basingstoke, Hampshire, United Kingdom) for at least 5 days, and colony counting was performed after this period of time.

### Antibiofilm Activity of Nisin

The activity of nisin on the formation of biofilm by two LAB strains of enological origin (*O. oeni* IS151 and *Leuconostoc mesenteroides* J32) was assessed. These two strains were selected because they showed the highest biofilm-forming activity during a previous screening for biofilm-forming activity among the enological LAB strains of our collection. A bacterial suspension of a fresh culture was grown in Brain-Heart Infusion Broth (BHI, Becton Dickinson, Sparks, MD, United States) without and with 6 μg/mL nisin (Sigma-Aldrich) and either in absence or in presence of ethanol (2, 4, 6, and 8% v/v). Samples were incubated at 23°C without agitation for 48 h to allow biofilm formation. Assays were performed in triplicate. Bacterial growth was determined by colony counting on MRS-agar plates, as described above. Biofilm mass was evaluated by the crystal violet staining assay (O’Toole and Kolter, 1998) and values above 1 of optical density (OD) at 600 nm were considered indicative of positive biofilm formation. These experiments were repeated on a 48 h pre-formed biofilm and addition of fresh culture broth containing binary combinations of nisin (0, 6, 200 μg/mL) and ethanol (0, 2, 4, 6, 8% v/v). Incubations were maintained for 24 h, after which the biofilm mass was evaluated by the crystal violet assay.

### Nisin Production by *L. lactis* LM29 Under Enological Conditions

Nisin production by *L. lactis* LM29 was tested as previously described (Rojo-Bezares et al., 2007a), using *Pediococcus pentosaceus* FBB63 as the indicator strain. The nisin producer *L. lactis* LM29 was grown in MRS broth (Scharlau Chemie S.A.) containing a range of concentrations of filter-sterilized white grape must (0, 20, 40, 60, and 80% v/v) and adjusted to pH 3.5. It was also incubated in MRS broth with ethanol (Panreac Química, S.A.) (0, 2, 4, 8, and 12% v/v) and in a combination of 60% must and ethanol (0, 2, and 4%). In all these experiments culture broths were adjusted to pH 3.5, the initial cell concentration of *L. lactis* LM29 was 10^4^–10^7^ CFU/mL, and samples were incubated for 48 h at 22°C without agitation. Samples were taken at different times during bacterial growth to estimate bacterial population by OD at 660 nm, and also nisin activity of the cell-free supernatants. Samples for the assay of nisin activity were maintained in a boiling water bath for 15 min and then spun (Heraeus Biofuge, Thermo Scientific, Wilmington, DE, United States) at 14,800 × *g* for 5 min; cell pellets were eliminated and supernatants were collected in a clean tube for further analysis of antimicrobial activity by the agar diffusion and microtiter methods as previously described (Rojo-Bezares et al., 2007a). Antimicrobial activity of all samples was assayed in duplicate. In all cases a control sample was included, which was prepared with the culture broth without inoculation of *L. lactis* LM29.

After determining optimal conditions for *L. lactis* LM29 growth and nisin production, a volume of 45 mL of cell free supernatant was prepared by growing *L. lactis* LM29 for 30 h in MRS broth containing 60% white grape must and following the steps described above to obtain a cell-free extract. The concentration of nisin in this active extract was calculated by comparison of its activity with a pure nisin standard (Sigma-Aldrich Chemie) in the microtiter assay against the indicator strain *P. pentosaceus* FBB63. This nisin enriched active extract (NAE) was tested against the collection of 108 LAB and AAB strains included in **Table 1**. One arbitrary unit of antimicrobial activity (AU) was defined as the maximum dilution factor of the active extract that inhibited 50% of the strain growth. The minimal inhibitory concentration MIC_50_ value was defined as the concentration of nisin in the active extract that inhibited 50% of the tested strains.

The growth of *L. lactis* LM29 in presence of ethanol was also studied by fluorescence microscopy using the kit of fluorescence for bacterial viability Live/Dead BacLight (Molecular Probes, Eugene, OR, United States) and observation under the fluorescence microscope Axioscope 2 Plus (Zeiss, Madrid, Spain), following the method previously reported (Fernández-Pérez et al., 2010). This method allowed us to determine viable cells (stained with Syto 9 that fluoresce green) and non-viable cells with damaged cytoplasmic membranes (stained with propidium iodide that fluoresce red).

### Statistical Analysis

Analysis of variance (ANOVA) was used to evaluate significant differences. IBM-SPSS Statistics 22.0 software for Windows (IBM-SPSS Inc., Chicago, IL, United States) was used for data processing.

## Results and Discussion

### Combined Effect of Nisin and Sulfur Dioxide on Bacterial Growth in Wine

**FIGURE 1:**
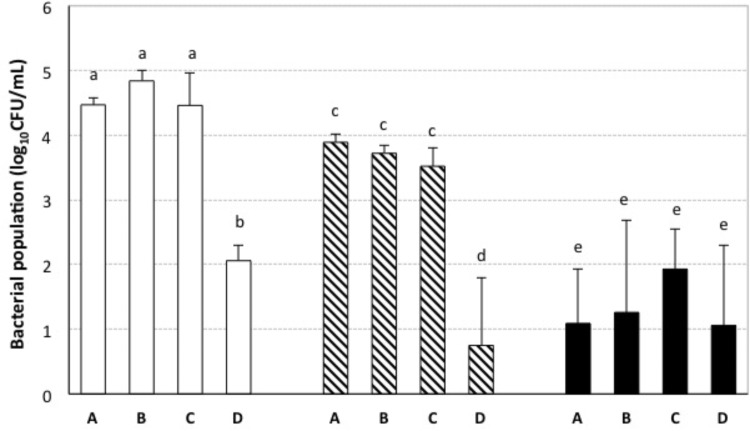
LAB populations in wines submitted to aging with addition of different combinations of nisin and potassium metabisulphite. Bacterial populations (log_10_CFU/mL) were measured after 3 months of aging. Potassium metabisulphite concentrations: 18 mg/L; 25 mg/L; 100 mg/L nisin concentrations: A = 0 mg/L, B = 0.78 mg/L, C = 6.25 mg/L, and D = 50 mg/L. Different letters (a, b, c, d, e) indicate significant differences (*p* < 0.05; variable parameter: nisin concentration) among data obtained for the wines treated with the same concentration of potassium metabisulphite.

A previous study of our research group had shown that nisin either alone or in combination with sulfur dioxide inhibited the growth of enological LAB cultures under laboratory conditions (Rojo-Bezares et al., 2007b). In the same study, the minimal inhibitory and bactericide concentrations of nisin in culture broths had been determined. Some reports have suggested that the inhibitory activity of bacteriocins in culture broths is not always reproducible in food matrices since their activity may be affected by interactions with additives and food components (Collins et al., 2011). Therefore, in order to investigate whether the inhibitory effect of nisin could be applied to wine-making, we elaborated Tempranillo red wines that were submitted to aging with addition of different combinations of nisin and potassium metabisulphite, and which contained a high initial LAB population before additions (log_10_[CFU/mL] = 4.4 ± 0.1). The results of the microbiological analyses of our wines after 3 months of aging are shown in **Figure 1**. The populations of AAB in these wines were not affected by the presence of nisin in the concentrations that were used, and population values were very low (log_10_[CFU/mL] <1) in all cases. After 3 months of aging, the wines with low concentrations of potassium metabisulphite (18 or 25 mg/L) and with nisin at a concentration of 50 mg/L presented a statistically significant reduction in their LAB populations (log_10_[CFU/mL] <2) (**Figure 1**). These results indicate that the use of nisin allowed a fourfold reduction in the concentration of potassium metabisulphite required to prevent LAB growth during the studied period of wine aging. These results are in agreement with other results reported for wines elaborated with red grapes (Daeschel et al., 1991; Neris et al., 2013) and tangerines (Pei et al., 2016), which also showed a decrease of LAB populations in the wines when nisin was added in combination with sulfur dioxide. It should be noted that an acidic pH, such as that of wines (3 < pH < 4), can potentiate the inhibitory activity of nisin (Silva et al., 2018) as it increases the net charge and solubility of nisin and facilitates its diffusion and translocation through the cell wall (Gálvez et al., 2007). However, the addition of nisin to wine as an additive or preservative is not currently approved for wine-making.

### Effect of Nisin on LAB Biofilm Formation

**FIGURE 2:**
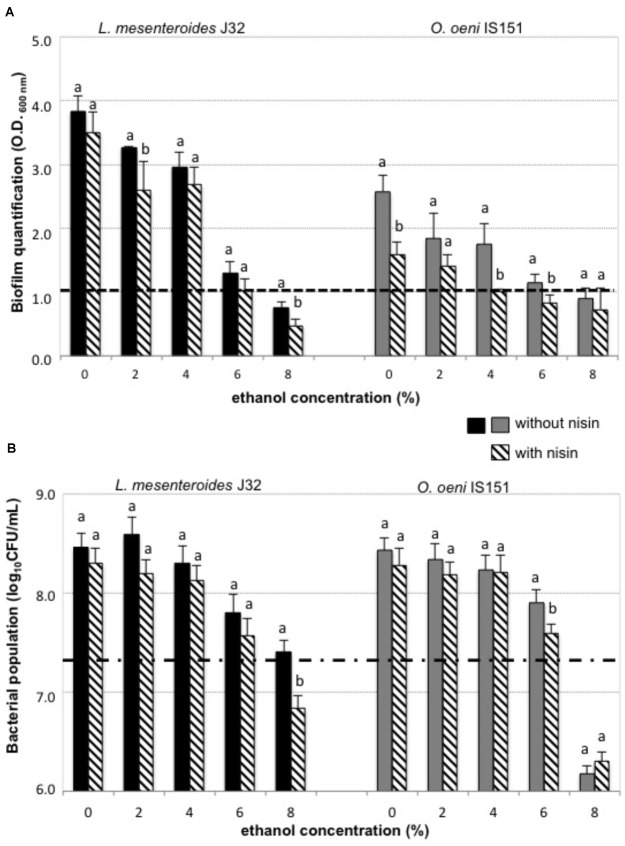
Biofilm formation and bacterial growth in presence of nisin and ethanol in the culture broth of planktonic cells, **(A)** biofilm was quantified after 48 h incubation without nisin (solid color bars) or with 6 μg/mL nisin (diagonal striped bars) in the culture broth and with (0–8%) ethanol: dashed line (1 a.u.): above which it is considered positive biofilm formation, **(B)** bacterial growth was quantified after 48 h incubation without nisin (solid color bars) or with 6 μg/mL nisin (diagonal striped bars) in the culture broth and with (0–8%) ethanol; dashed line: initial bacterial population above which it is positive the bacterial growth of *L. mesenteroides* J32 (black bars) and *O. oeni* IS151 (gray bars).

Given the resistance of enological LAB strains to the acid pH of wine and that it has been proved that biofilm formation is an important strategy for microbial survival under acid stress conditions (Wang et al., 2018), we investigated the biofilm-forming ability of two selected enological LAB strains that showed a biofilm positive phenotype. The results of these experiments, performed with combinations of nisin (6 μg/mL) and a range of ethanol concentrations, are shown in **Figure 2A**. In all cases the presence of ethanol in the culture broth reduced the amount of formed biofilm, as well as the bacterial growth. This inhibitory effect of ethanol is in agreement with the model for biofilm formation by Gram-positive bacteria (Vlamakis et al., 2013), where ethanol may exert its inhibitory activity at two levels: (1) acting as a biofilm disruptor (Böttcher et al., 2013) by interacting with the exopolysaccharides produced by planktonic bacteria to form the biofilm, and thus, disrupting the required aggregation for biofilm formation; and (2) as a bactericide molecule, provoking bacterial death, and, consequently, decreasing the density of viable cells, which results in retarding or preventing the formation of biofilm. The mechanisms of ethanol stress in *O. oeni*, and its molecular response are well known and have been previously reported (Bonomo et al., 2018), whereas to our knowledge, this is the first report describing the response of *L. mesenteroides* to ethanol.

**FIGURE 3:**
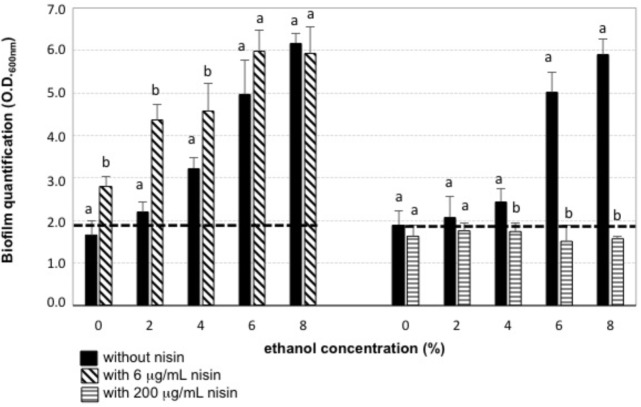
Addition of fresh culture broth containing binary combinations of nisin and ethanol on a preformed bacterial biofilm. The preformed biofilm of *L. mesenteroides* J32 in absence of antimicrobials, was quantified after 24 h incubation with fresh culture broth containing: 0 μg/ml nisin (black bars), 6 μ/mL nisin (diagonal striped bars), 200 μg/ml (horizontal striped bars) in binary combinations with ethanol (0–8%); dashed line: initial preformed biofilm.

Both biofilm-forming strains, *O. oeni* IS151 and *L. mesenteroides* J32, were able to generate their biofilms in presence of 4 and 6% ethanol, respectively (**Figure 2A**); in contrast, the strains were no longer able to form biofilms when nisin (6 μg/mL) had been added to the culture broth. The inhibitory effect of nisin on the growth of these LAB strains in their planktonic form is shown in **Figure 2B**. These results indicate that nisin was able to reduce the capacity of both strains to generate their biofilms, and that in combination with ethanol (4–6%) a nisin concentration of 6 μg/mL was able to prevent the formation of biofilms. These results are in agreement with recent studies that indicate that antimicrobial peptides, such as nisin, can act as biofilm disruptors due to their ability to permeabilize bacterial membranes (Smith et al., 2016; Grassi et al., 2017). Nevertheless, when we performed the experiments of addition of the culture broth containing binary combinations of 6 μg/mL nisin and ethanol (0, 2, 4, 6, 8% v/v) on a previously formed biofilm, instead of on planktonic cells of *O. oeni* IS151 and *L. mesenteroides* J32, the results were completely different: in all cases the pre-formed biofilms enlarged after 24 h incubation with the culture broth containing the subinhibitory concentrations of both nisin and ethanol (**Figure 3**). Only when the nisin concentration was increased to 200 μg/mL (well above its minimal inhibitory concentration of 12.5 μg/mL) biofilms decreased significantly after 24 h incubation. These results are in agreement with the fact that biofilm formation constitutes a survival strategy that protects the integrity of the microbial cells against harsh conditions and antimicrobial agents (Wang et al., 2018); in addition, exposure of biofilm cells to subinhibitory concentrations of an antimicrobial agent can induce the expression of a variety of genes related to drug resistance, such as efflux pumps or synthesis of exopolysaccharides, thus activating the resistance mechanisms of bacteria by inducing and enlarging biofilm structures (Vlamakis et al., 2013; Singh et al., 2017). In this regard, the effect of sublethal concentrations of ethanol that increase the formation of biofilms of Gram-positive bacteria has been studied in pathogenic bacteria of the genus *Staphylococcus* (Cincarova et al., 2016; Slany et al., 2017; Wang et al., 2017). In relation to the enological species of our study, the production of exopolysaccharides by *L. mesenteroides* and *O. oeni* enological strains (Ibarburu et al., 2007; Montersino et al., 2008) and other LAB species of enological origin (Garai-Ibabe et al., 2010) has been reported, nevertheless, to our knowledge there are no previous reports on the effect of ethanol on the formation of biofilms by these enological species. Our results can be explained by the biofilm forming mechanism proposed for Gram-positive bacteria, where the antimicrobial molecule is able to penetrate the cellular membrane leading to the efflux of potassium ions and a cascade of signaling which activates the central transcriptional regulator that controls the expression of a range of genes, including those necessary for the biofilm matrix formation and the subsequent biofilm maturation (Vlamakis et al., 2013). With respect to nisin, to our knowledge this is the first report on the activation of biofilm synthesis by a subinhibitory concentration of this bacteriocin. Previous studies reported the inhibitory effect of nisin on the growth and biofilm formation of pathogenic bacteria, such as *Listeria* (Smith et al., 2016), *Staphylococcus* (Dosler and Mataraci, 2013; Okuda et al., 2013; Field et al., 2016a), *Streptococcus* (Tong et al., 2014), *Enterococcus* (Taneja et al., 2015), *Bacillus* and *Salmonella* (Bag and Chattopadhyay, 2017), *Bacillus* and *Clostridium* spores (Egan et al., 2016), *Pseudomonas* (Field et al., 2016b), and *Escherichia coli* (Al Atya et al., 2016), but none of them reported any biofilm activating effect by subinhibitory concentrations. Regarding *O. oeni*, a biocide effect of nisin on *O. oeni* cells in biofilms formed on stainless steel surfaces was already reported (Nel et al., 2002). Our results indicate that the effect of nisin in low concentrations (6 μg/mL) is just opposite to desired, as it promotes an increase of pre-formed biofilms independently of the absence or presence of ethanol, and demonstrate that nisin concentration should be raised to 200 μg/mL to combat pre-formed biofilms.

### Nisin Production by *L. lactis* LM29 Under Enological Conditions

**FIGURE 4:**
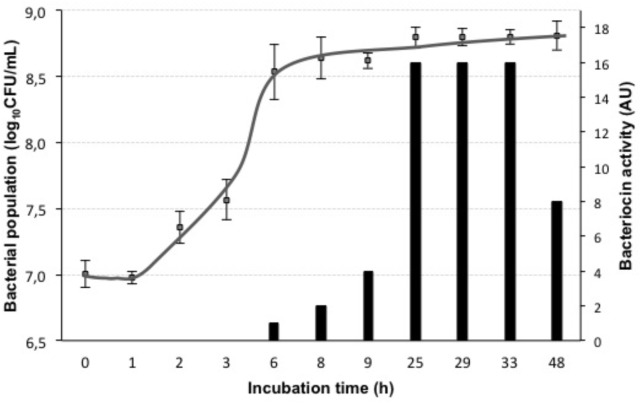
Growth curve and bacteriocin activity of *L. lactis* LM29. Bacterial population (log_10_CFU/mL) (gray squares) was measured over a period of 48 h incubation in MRS broth, Bacteriocin activity (AU) (black bars) was measured by the microtiter assay against the indicator strain *P. pentosaceus* FBB63.

**FIGURE 5:**
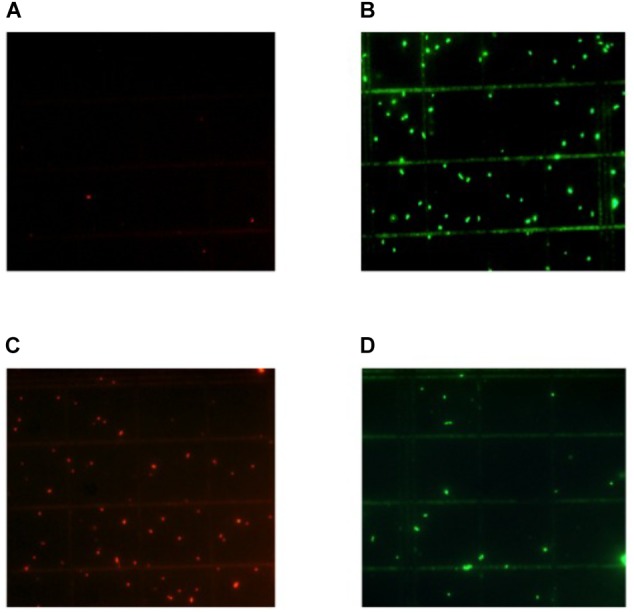
Fluorescence microscopy images of *L. lactis* LM29 after 24 h incubation either in presence or in absence of ethanol in the culture broth. **(A,B)** Cells incubated in MRS broth. **(C,D)** Cells incubated in MRS broth with 12% ethanol. **(A,C)** Non-viable bacterial cells, with damaged cytoplasmic membrane, stained with propidium iodide that fluoresce red. **(B,D)** Viable cells, with intact cytoplasmic membrane, stained with Syto 9 that fluoresce green.

**FIGURE 6:**
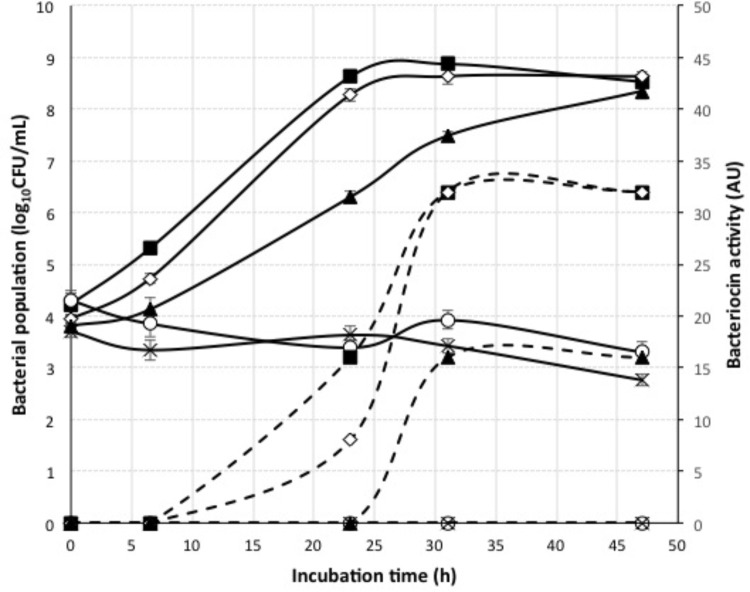
Growth curves and bacteriocin activity of *L. lactis* LM29 in presence of different ethanol concentrations. Bacterial population (log_10_CFU/mL) (continuous line) was measured over a period of 48 h incubation. Bacteriocin activity (AU) (dashed line) was measured by the microtiter assay against the indicator strain *P. pentosaceus* FBB63. Ethanol concentrations: 0, 2, 4, 8, 12%.

The application of nisin for biopreservation may be performed following several strategies: (a) adding the purified or semi-purified nisin as preservative; (b) adding a product previously fermented with the nisin-producing strain; and (c) inoculating the nisin-producing strain in the fermenting food or beverage. To investigate the possibility of applying the last two strategies to wine-making, we cultivated the nisin-producing strain *L. lactis* LM29 in a variety of culture broths resembling enological compositions. **Figure 4** shows the growth curve of this strain and its production of nisin in MRS broth, which started during *L. lactis* LM29 stationary growth phase. A range of concentrations of white grape must (0, 20, 40, 60, and 80% v/v) (data not shown) and ethanol (0, 2, 4, 8, and 12% v/v) were assayed (**Figure 6**). **Figure 5** shows the fluorescence microscopy images of *L. lactis* LM29 after 24 h incubation in MRS broth and in MRS broth with 12% ethanol. Non-viable bacterial cells with damaged cytoplasmic membrane fluoresce red (**Figure 5C**) and can be observed in the sample treated with ethanol. Similarly, **Figure 6** shows that *L. lactis* LM29 was able to grow in presence of up to 4% ethanol but not with 8% or higher ethanol concentrations, and in addition, nisin production decreased progressively as ethanol concentration increased. This result was in agreement with another study that revealed that *L. lactis* transcriptomic response to the presence of ethanol in the culture broth induced repression of the *nis* operon responsible for nisin production (Díez et al., 2017). Conversely, in our assays *L. lactis* LM29 was able to grow and produce nisin in presence of grape juice up to 60% v/v without any inhibitory effect. Therefore, the extract we called NAE was prepared and this extract showed high activity both in the agar diffusion test (2 cm diameter of inhibition halo) and in microtiter assays. When the activity of NAE against the indicator strain *P. pentosaceus* FBB63 was compared with the activity of a pure nisin standard, it showed an equivalent activity of 200 μg nisin/mL. Then the activity of NAE was tested against the collection of 108 LAB and AAB strains of enological and vinegar origin, and **Table 2** shows the results of this screening of susceptibility to the antimicrobial activity of NAE. As previously reported (Rojo-Bezares et al., 2007b) *O. oeni* was clearly the most susceptible species to nisin (*p* < 0.001), while the other enological LAB strains showed an intermediate MIC_50_ value (4 AU, equivalent 12.5 μg nisin/mL). AAB were the most resistant bacteria (>50 μg/mL), which was also in agreement with the previous report (Rojo-Bezares et al., 2007b) and with the fact that nisin is regarded as an inhibitor of Gram-positive bacteria (Field et al., 2015). Actually, some authors have proposed the use of nisin to eliminate Gram-positive beer spoilage bacteria in breweries (Müller-Auffermann et al., 2015). Our results in wine also corroborate this inhibitory effect of nisin on the growth of LAB in the complex matrix of wine and in combination with metabisulphite. Interestingly, in our screening of susceptibility to nisin (**Table 2**) we found eight *O. oeni* strains that showed resistance, and therefore, they could constitute excellent candidates for selection in combination with nisin for starter cultures for MLFs of red wines to achieve an efficient microbiological control of the process. Moreover, the obtained nisin-enriched fermented extract could also provide a safe partial replacement of sulfur dioxide and contribute to reducing the concentrations of this chemical preservative that is currently added to musts and wines in wine-making.

**Table 2:** Antimicrobial activity of the nisin active extract (*L. lactis* LM29) against enological LAB and AAB (wine and vinegar origin) strains by the microtiter assay.

Antimicrobial Activity (AU^∗^)	Number of *O. oeni* strains (total: *n* = 38)	Number of LAB^∗^ strains (total: *n* = 41)	Number of AAB strains (total: *n* = 29)
0	8	5	21
*1*	0	6	5
*2*	1	7	1
*4*	0	8	0
*8*	1	7	0
*16*	1	4	1
*32*	2	1	1
*64*	2	1	0
*128*	13	1	0
*256*	3	1	0
*512*	3	0	0
*1024*	0	0	0
*2056*	4	0	0

*AU, arbitrary units; LAB^∗^, lactic acid bacteria except O. oeni. One AU is defined as the maximum dilution factor of the active extract that inhibits 50% of the strain growth. Italic values correspond to the Antimicrobial Activity of the nisin active extract, in its units (AU)*.

## Author Contributions

All authors listed have made a substantial, direct, and intellectual contribution to the work, and gave the final approval for publication.

## Conflict of Interest Statement

The authors declare that the research was conducted in the absence of any commercial or financial relationships that could be construed as a potential conflict of interest.
